# Another crisis in the sorrowland: COVID-19 in northeast Syria

**DOI:** 10.7189/jogh.12.03033

**Published:** 2022-08-31

**Authors:** Rebecca Forman, Lorenzo Ciancaglini, Pedro San Jose Garces, Michailia Neli, Elias Mossialos

**Affiliations:** 1 LSE Health, London School of Economics & Political Science, London, UK; 2 Relief International; 3 Un Ponte Per, North East Syria, Syrian Arab Republic; 4 Institute of Global Health Innovation, Imperial College London, London, UK

While for many countries, the management of COVID-19 has been well-documented, less is known about the response efforts in war and conflict-stricken areas such as North-East Syria (NES).

NES was forced to confront SARS-CoV-2 amidst a slew of existing crises [1]. As it reaches its tenth year, the Syrian conflict remains one of the largest, longest, and most complex humanitarian emergencies, and estimates suggest that of the five million people in acute need of humanitarian assistance in Syria, 1.4 million reside in NES – the enclave run by the Autonomous Administration (AA) and controlled by the Syrian Democratic Forces [2].

Though the AA largely has *de facto* political and operational autonomy over NES, the region is neither recognised officially by the Government of Syria (GoS) nor by any state or international organisation [3]. This creates challenges, since NES has its own unique structures, leadership, governance, and funding [3]. Even before conflict in Syria began, the health system in NES was fragile from years of underinvestment [1]. The UN Security Council’s January 2020 suspension of cross-border assistance to NES exacerbated existing challenges, and the additional strain from the COVID-19 pandemic weakened the system further. A June 2021 Open Letter to the United Nations (UN) Security Council signed by eminent leaders of world-renowned organisations including the International Rescue Committee, Save the Children, CARE International, and Oxfam International, stated that 69% of people in NES need aid, half of which are children [4]. Furthermore, the needs in NES have grown by 38% since the crossing closed [4].

## BUILDING A COVID-19 DEFENCE WITH LIMITED RESOURCES

Even against this challenging political and social backdrop, valiant efforts have been made to prevent and respond to COVID-19 infection spread in NES since its emergence. In early March 2020, the AA and the humanitarian sector began discussing COVID-19 prevention measures, and in April and May 2020, a Technical Committee (TC) of experts from various NGOs was established to advise the Department of Health (DoH), the central health authority of the AA.

Since then, under the guidance and expertise of the TC, and in coordination with other health sector actors, COVID-19 committees composed of health officials and NGO officers were established at the local level in NES to engage with communities, improve risk communication efforts, deploy contact tracers and community mobilisers, strengthen epidemiological surveillance systems, and improve infection and prevention measures – both in health facilities and in public spaces. Extensive efforts were also made to prevent widespread infection in refugee camps and informal settlements (see **Box 1**). Additionally, the AA opened a laboratory with testing equipment and isolation facilities for moderate and severe cases of COVID-19. At least 26 rapid response teams for testing and referral were deployed in the region in 2020 since the pandemic began, and a hotline system with an operation desk for referral was established [11]. Travel in and out of NES was also heavily restricted for a period in 2020 [12].

Box 1 **Managing covid-19 in refugee camps and informal settlements**

Recognising that refugees and internally displaced persons (IDPs) in camps and informal settlements have some of the greatest risks for infectious disease spread, isolation areas for suspect and confirmed cases within the camps were prepared and dedicated vulnerability mapping and follow-up were conducted by community health workers early on in the pandemic response [5].

At the time of writing, the strict COVID-19 response measures in camps appeared to limit transmission – a glimmer of light in otherwise extremely trying circumstances. As of June 2021, there were no outbreaks in these areas and only 1.7% of confirmed cases had been registered in camps or informal settlements [6]. By the end of 2021 there had not been any major outbreaks in refugee camps around the world [7]. A multitude of factors have likely influenced this outcome, including early implementation of lockdowns in the camps and the greater ease of enforcing these restrictions in camp settings, limiting outsiders’ access into the camps, low average age of camp residents, and effective epidemiological surveillance [7]. However, there is an undersupply of PCR tests in NES (and in many places with refugee camps around the world), and thus, true rates of infection are likely to be higher than those reported.

Nonetheless, COVID-19 infection is not the only threat to health and well-being faced in camps and informal settlements. Evidence suggests that COVID-19 has had severe compounding effects on existing inequalities. Rates of food insecurity and hunger, unemployment, anxiety and social isolation are on the rise and threaten the social fabrics holding refugee communities together as violent conflicts persist [8-10]. This may have long-term effects far beyond the pandemic period.

## INFECTION SPREAD

Aside from six COVID-19 cases in April 2020, NES saw no further documented cases again until the end of July 2020 [6,13]. This could have been attributed to limited testing and surveillance capacity in the region and other related challenges at the time. However, in the Summer of 2020, with illegal crossings between borders and the return of thousands of students from exam sessions in Damascus, unrelated COVID-19 infection clusters emerged in NES, after which situation quickly spiralled out of control.

Registered total COVID-19 case numbers since the start of the pandemic jumped from 101 cases in the first week of August 2020 [11] to 841 by early September 2020 [14]. While estimates of the total population in NES vary widely across sources, WHO Regional Office for the Mediterranean’s estimates of 2.6 million people in NES [15] would mean this was an increase from 3.88 cases per 100 000 people to 32.35 cases per 100 000 people in just a month. The AA implemented a ten-day partial lockdown (which had long been advocated for by the TC), awareness campaigns, school closures, and curfews [11]. Travel restrictions within governorates were also instated. Due to the militarised context and existing checkpoints in the area and the lack of public transport, these measures were mainly abided by.

Despite these efforts, from October 1 to November 10, 2020, the cumulative total number of confirmed cases in NES more than tripled (from 1708 to 5827), pushing far beyond the health system’s capacity [6]. Early in November 2020, there were only 491 beds for moderate and severe COVID-19 cases in the region, leaving a gap in planned coverage of ICU beds at 81% [16]. Even now, there are no more than 800 beds for moderate and severe cases, and coverage varies between different districts. In Spring 2021, NES faced a steep second wave; in late 2021, it faced its worst wave yet, with severely limited oxygen and testing supplies; this was followed by another smaller wave in early 2022 [6].

**Figure:**
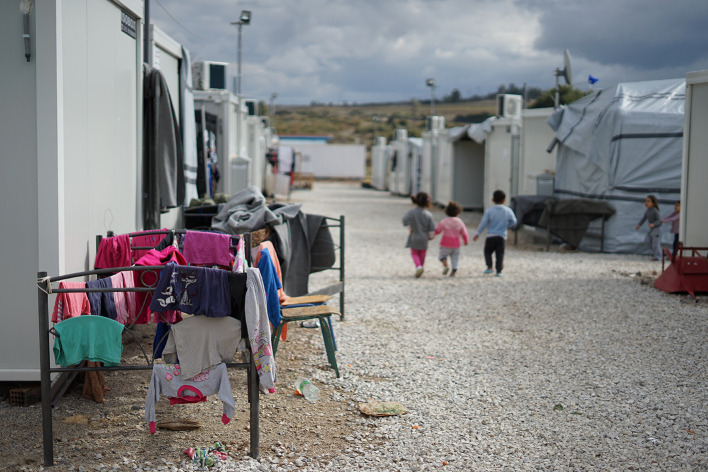
Photo: Syrian refugee camp in the outskirts of Athens. Over 6.6 million Syrians were forced to flee their home since 2011. Many are still stranded in refugee camps in Turkey and Greece, waiting (and hoping) to be granted asylum in European countries or beyond. According to the United Nations Universal Declaration of Human Rights of 1948, “everyone has the right to seek and to enjoy in other countries asylum from persecution”. In practice, despite the existence of well-founded fear of persecution, many host countries are not upholding the basic right of asylum. (Source: Julie Ricard, via https://unsplash.com/photos/MX0erXb3Mms. No permission needed).

From March 22, 2020, to February 26, 2022, there had been 38 394 PCR-confirmed COVID-19 cases in NES and 1552 confirmed COVID-19 deaths, bringing the official case fatality rate in NES to 4% [17]. However, given the testing and tracing capacity issues mentioned above and the lack of access to health care services in the region, these figures are likely underestimates of the true extent of the COVID-19 outbreak in NES [18]. In December 2021, the number of reported cases declined significantly because of laboratory testing interruptions and struggles in securing consistent support for the Qamishli Central laboratory [19]. However, reporting with limited capacity resumed in January 2022 [20]. The 916 new confirmed COVID-19 cases in NES in February presented a 217% increase from the 289 confirmed cases in the previous month [18]. Despite this increase in positive cases, the number of COVID-19 deaths declined during the same period. Notably, as of September 2021, over 1206 of the estimated total 8900 health care workers (13.6%) in NES had experienced confirmed COVID-19 infection [21]; creating further consequences for the health system.

## COVID-19 VACCINATION CAMPAIGNS UNDERWAY BUT FACE UNDERSUPPLY, INSUFFICIENTLY TRAINED MEDICAL STAFF, VACCINE HESITANCY, AND MORE

A multitude of challenges related to the COVID-19 vaccines exists from development to dissemination, through to deployment [22], particularly in places such as NES, which already faced resource constraints, lack of infrastructure, human resource capacity, and more. In May 2021, the Syrian government delivered 22 500 of the 203 000 Astra Zeneca Vaxzevria vaccine doses it received through COVAX to NES [23] so that NES could begin rolling out COVID-19 vaccinations as part of the National Immunisation Programme. Vaccines shipped to NES were sufficient to cover all health care workers in NES, a select number of people aged 55 and above, and people with co-morbidities (with two doses), who were initially among the vaccination priority groups [24]. However, considering their population sizes, this first COVAX shipment was only enough to give one jab to about 1% of those in Syria, and less than 1% in NES.

Initially, communication and coordination challenges between the various health authorities in NES hindered efforts. The WHO, based on its experience successfully supporting the national immunisation campaign in Syria, mediated between stakeholders and provided operational and technical support so that vaccination rollout could begin at a limited number of fixed sites in Al-Hasakeh on 18 May, then expand onto fixed and mobile vaccination teams deployed in the governorate, and then to drives in Ar-Raqqa, Deir-ez-Zor, Aleppo in early June [24]. NES began administering the second batch of 9992 vaccines from COVAX in September 2021 [23]. In addition to the AZ Covishield, AZ/AZD1222, Sinovac, and Janssen vaccines obtained via COVAX, at the time of writing, the Syrian government had also secured doses of AZ/AZD1222, Sinopharm, Sputnik light, Sputnik V, and Soberana through bilateral agreements, some of which have been delivered to NES to vaccinate residents and health care staff [17].

By late June 2021, 11 012 people in NES had received their first jabs, including 2222 health care workers and 8790 people with comorbidities or were aged 55+; the second batch had reached a further 7894 people as of early September 2021 [23]. Still, vaccine supply in NES, and in broader Syria, is hugely inadequate and many challenges, including vaccine hesitancy [25], remain in the efforts to increase vaccination rates (see **Box 2**). As COVID-19 cases surged in NES and broader Syria in the Autumn of 2021, vaccination rates could not keep up [19]. As of February 2022, the WHO reported that less than 11% of people in the whole of Syria had received at least one dose, and only about 6% had been fully vaccinated [17]. The weekly average of administered vaccine doses fell by 27% in Syria from January to February 2022 [17]. Teams on the ground must balance efforts to generate demand and decrease vaccine hesitancy while also promoting realistic expectations about vaccine availability and the health system’s capacity to deliver vaccines.

Box 2 **Campaigns hit by vaccine hesitancy [26] – even among health care workers initially**

With the limited health system capacity, the high proportion of positive COVID-19 cases among health care workers, and the vital role that they play in tackling the pandemic, health care workers were the top priority group for vaccine campaigns. The vaccine doses allocated to NES in the first COVAX delivery were sufficient to cover 100% of its health care workers, but uptake, even amongst those on the front lines, was initially an issue due to vaccine hesitancy [23]. WHO, UNICEF, and other partners on the ground (with generous support from others – including Sweden) have taken a proactive approach to try to address the public’s concerns and have developed a risk communication and community engagement (RCCE) strategy, which prioritises camps and informal settlements [24,27]. RCCE messages under the AA have intensified, and with these efforts, greater availability of vaccines, and more people receiving jabs without severe side effects, there has been gradual progress in reducing hesitancy [23]. Seeing that segments of the health care workforce were refusing vaccines early in the campaign, other high-risk groups were added to the vaccine allocation priority list to avoid wasting doses.

## REMAINING CHALLENGES

While essentially all countries around the world have struggled to manage the COVID-19 pandemic and have learned lessons in the process [28], many of the gaps in preparation and implementation that have contributed to the rapid spread of COVID-19 and the challenging start of vaccination efforts in NES can be linked to the lack of internal and international resources and support for health in the region that existed long before the pandemic hit. The health system already suffered from the consequences of ongoing conflict, violence, and political strife [1,29]. This made it almost impossible for the AA health authorities to attain the key qualities for building a successful COVID-19 response: strong governance, reliable and centralised information systems, a pervasive referral system, and human resource capacity. Furthermore, no fully functional facilities (defined by WHO as open, accessible, and providing health care services with full capacity – staffing, equipment, and infrastructure) are available in NES [30]. NES also struggles with several water, sanitation, and hygiene (WASH) challenges, which present even more difficulties in containing an infectious illness like COVID-19. High rates of food insecurity and impacts of violent conflict on mental health, education, and job security that have long been prevalent in NES, and Syria more broadly, have been exacerbated by the pandemic [19].

On top of this chronic under-resourcing and lack of support for health in NES, the UN Security Council suspended the delivery of humanitarian aid by UN agencies from Iraq to NES in January 2020 [19]. This led to further reductions in funding for the health sector, weakened food assistance programmes, and hindered access to critical medical supplies and humanitarian assistance [31]. While WHO and other agencies have continued some crossline health supply deliveries after the January 2020 suspension, this is insufficient to meet all health supply needs; there is still a large undersupply of medical equipment, including PCR tests [32].

With the ongoing conflict and the severe human and financial resource constraints, countering SARS-CoV-2 in NES has been very difficult, more so as it is hard to know what the true COVID-19 spread is in the region. The health information system is not unified, let alone digitalised, and has been unable to track the evolution of the pandemic and the response. Furthermore, some of the local COVID-19 committees do not follow DoH guidelines, and as a result, they have created competing hotlines and referral mechanisms, confusing the contact tracing processes and minimising its effectiveness. The dearth of vaccines available to the region also presents a major challenge in the fight against COVID-19.

The COVID-19 pandemic has highlighted how humanitarian interventions in Syria are shaped by the strategic interests of external actors, and how this has created consequences for NES. It also demonstrates that in war- and conflict-stricken areas, it is important that the humanitarian system is empowered to navigate political difficulties and cooperate with local health authorities, which can provide essential health services in emergency circumstances.

The situation in NES also starkly highlights the vast global inequities in access to basic health resources. By February 2022, many larger and wealthier countries had already administered booster shots to their populations and begun to lift restrictions; meanwhile, NES did not have enough jabs to fully vaccinate even 10% of its population at the time, and the WHO target of 70% vaccination in all countries by mid-2022 was far from reach [23]. This needs to change; not only for ethical reasons, but also because we cannot defeat COVID-19 and its variants until there is adequate protection across the entire world.

## References

[1] Bery S Ciancaglini L Garces PSJ Brim B Wharton G Mossialos E Time to address the plight of the people of northeastern Syria. Lancet. 2019;393:1394-6. 10.1016/S0140-6736(19)30491-X 30967201

[2] UN Office for the Coordination of Humanitarian Affairs. 2019 Humanitarian Needs Overview: Syrian Arab Republic. Available: https://reliefweb.int/report/syrian-arab-republic/2019-humanitarian-needs-overview-syrian-arab-republic-enar Accessed: 17 June 2021.

[3] Abbara A. COVID-19 Exposes Weaknesses in Syria’s Fragmented and War-Torn Health System. MERIP. 2020. Available: https://merip.org/2020/12/covid-19-exposes-weaknesses-in-syrias-fragmented-and-war-torn-health-system/. Accessed: 26 August 2021.

[4] CARE, Christian Aid, Concern Worldwide, Handicap International - Humanity & Inclusion, InterAction, International Rescue Committee, MedGlobal, Mercy Corps, Mercy-USA for Aid and Development, Norwegian Refugee Council, Oxfam, People in Need, Refugees International, Relief International, Save the Children, Syria Relief, Syrian American Medical Society Foundation, Trócaire, War Child International, World Vision. Open Letter to United Nations Security Council Ambassadors regarding the upcoming vote on the Syria cross-border resolution - Syrian Arab Republic. ReliefWeb. Available: https://reliefweb.int/report/syrian-arab-republic/open-letter-united-nations-security-council-ambassadors-regarding. Accessed: 26 August 2021.

[5] Sites and Settlements NES Working Group. Monthly Update: North East Syria (June). REACH. 2020. Available: https://reliefweb.int/sites/reliefweb.int/files/resources/REACH_SYR_Factsheet_Informal-Site-and-Settlement-Profiles-RaqqaDZMenbij__June2020.pdf. Accessed: 17 June 2021.

[6] Forum NES. Northeast Syria COVID-19 Dashboard. Available: https://app.powerbi.com/view?r=eyJrIjoiNjA2ZDU0YmUtYWQyNS00YTBjLTg4YTctMjFhMDViZTc3Y2JjIiwidCI6ImY2ZjcwZjFiLTJhMmQtNGYzMC04NTJhLTY0YjhjZTBjMTlkNyIsImMiOjF9&pageName=ReportSectione26b6e9a7a7639c25b99. Accessed: 5 July 2021.

[7] Ndagijimana VN, Guensburg C. Have Refugee Camps Escaped Mass COVID Infections? VOA. 2021. Available: https://www.voanews.com/a/have-refugee-camps-escaped-mass-covid-infections-/6374703.html. Accessed: 4 March 2022.

[8] Ganot S. Study in Jordan Shows COVID-19’s Severe Impact on Displaced, Vulnerable Youth. The Media Line. 2022. Available: https://themedialine.org/by-region/study-in-jordan-shows-covid-19s-severe-impact-on-displaced-vulnerable-youth/. Accessed: 3 March 2022.

[9] Jones N Baird S Hamad BA Bhutta ZA Oakley E Shah M Compounding inequalities: Adolescent psychosocial wellbeing and resilience among refugee and host communities in Jordan during the COVID-19 pandemic. PLoS One. 2022;17:e0261773. 10.1371/journal.pone.0261773 35108293 PMC8809558

[10] UNRWA. Syria, Lebanon and Jordan Emergency Appeal 2022. UNRWA. 2022. Available: https://reliefweb.int/report/syrian-arab-republic/syria-lebanon-and-jordan-emergency-appeal-2022. Accessed: 3 March 2022.

[11] Forum NES. NES Forum: COVID-19 Update No. 15. 2020 Aug. Report No.: 15.

[12] Forum NES. NES Forum: COVID-19 Update No. 13. 2020 Aug. Report No.: 13.

[13] Forum NES. NES Forum: COVID-19 Update No. 8. 2020 May. Report No.: 8.

[14] Forum NES. NES Forum: COVID-19 Update No. 16. 2020 Sep. Report No.: 16.

[15] World Health Organization Regional Office for the Eastern Mediterranean. Statement by WHO’s Regional Director for the Eastern Mediterranean on the 10th year of the Syria crisis. World Health Organization. 2021. Available: http://www.emro.who.int/media/news/statement-by-whos-regional-director-for-the-eastern-mediterranean-on-the-10th-year-of-the-syria-crisis.html. Accessed: 30 August 2021.

[16] Forum NES. NES Forum: COVID-19 Update No. 18. 2020 Nov. Report No.: 18.

[17] World Health Organization Syrian Arab Republic. Monthly COVID-19 Bulletin: February 2022. World Health Organization; 2022. Available from: https://reliefweb.int/sites/reliefweb.int/files/resources/monthly_covid-19_bulletin-february_2022.pdf. Accessed: 4 March 2022.

[18] Fouad FM, Soares L, Diab JL, Abouzeid A. The political economy of health in conflict: Lessons learned from three states in the Eastern Mediterranean Region during COVID-19. Journal of Global Health. 2022 Feb 12. Available: https://jogh.org/2022/jogh-12-07001/. Accessed 4 March 2022. 10.7189/jogh.12.07001 PMC8836263 35198151

[19] OCHA. Humanitarian Needs Overview: Syrian Arab Republic. OCHA. 2022. Available: https://reliefweb.int/sites/reliefweb.int/files/resources/hno_2022_final_version_210222-2.pdf. 3 March 2022.

[20] World Health Organization Syrian Arab Republic. Monthly COVID-19 Bulletin: January 2022. World Health Organization. 2022. Available: http://www.emro.who.int/images/stories/syria/Monthly-COVID-19_Bulletin-January-2022.pdf?ua=1. Accessed 28 February 2022.

[21] World Health Organization. Syria COVID-19 Morbidity and Mortality Summary (SMMS). World Health Organization. 2021 Sep. Report No.: 11. Available: http://www.emro.who.int/images/stories/syria/smms_issue11_21_september_2021.pdf?ua=1. Accessed 9 November 2021.

[22] Forman R Shah S Jeurissen P Jit M Mossialos E COVID-19 vaccine challenges: What have we learned so far and what remains to be done? Health Policy. 2021;125:553-67. 10.1016/j.healthpol.2021.03.013 33820678 PMC7997052

[23] World Health Organization Regional Office for the Eastern Mediterranean. Update on COVID-19 vaccination in Syria, 22 September 2021. World Health Organization. 2021. Available: http://www.emro.who.int/syria/news/update-on-covid-19-vaccination-in-syria-22-september-2021.html.

[24] World Health Organization Regional Office for the Eastern Mediterranean. Update on COVID-19 vaccination in Syria. World Health Organization; 14 June 20221. Available: http://www.emro.who.int/images/stories/syria/COVID-19-vaccination-update_WHO_Syria_January-June-2021.pdf?ua=1. Accessed: 26 August 2021.

[25] de Albuquerque Veloso Machado M Roberts B Wong BLH van Kessel R Mossialos E The Relationship Between the COVID-19 Pandemic and Vaccine Hesitancy: A Scoping Review of Literature Until August 2021. Front Public Health. 2021;9: 74778. 10.3389/fpubh.2021.747787 34650953 PMC8505886

[26] Fahim K, Ali H. This besieged Syrian province escaped the worst of covid. Then vaccine skepticism crossed the border. Washington Post. 2021 Nov 2. Available: https://www.washingtonpost.com/world/2021/11/02/idlib-syria-coronavirus-covid-refugees/. Accessed: 1 March 2022.

[27] UNICEF. UNICEF with WHO starts awareness campaign in northeast camp about COVID-19. UNICEF. 2021. Available: https://www.unicef.org/syria/stories/unicef-who-starts-awareness-campaign-northeast-camp-about-covid-19. Accessed: 3 March 2022.

[28] Forman R Atun R McKee M Mossialos E 12 Lessons learned from the management of the coronavirus pandemic. Health Policy. 2020;124:577-80. 10.1016/j.healthpol.2020.05.008 32425281 PMC7227502

[29] World Health Organization. Health Sector Bulletin: February 2022. World Health Organization. 2022. Available: https://reliefweb.int/sites/reliefweb.int/files/resources/health_sector_bulletin_february_2022.pdf. Accessed: 4 March 2022.

[30] World Health Organization Regional Office for the Eastern Mediterranean. HeRAMS annual report Public Health Centres in the Syrian Arab Republic: January - December 2020: health resources and services availability monitoring system Syrian Arab Republic. Cairo. 2020 Dec. Available: https://vlibrary.emro.who.int/idr_records/herams-annual-report-public-health-centres-in-the-syrian-arab-republic-january-december-2020-health-resources-and-services-availability-monitoring-system-syrian-arab-republic/. Accessed: 17 June 2021.

[31] Care International. Over 1 million people at risk of hunger in Syria if cross-border aid resolution not renewed, say aid groups. Care International. 2021. Available: https://www.care-international.org/news/press-releases/over-1-million-people-at-risk-of-hunger-in-syria-if-cross-border-aid-resolution-not-renewed-say-aid-groups#_ednref1. Accessed: 17 June 2021.

[32] Forum NES. NES Forum: COVID-19 Update No. 21. 2021 Mar. Report No.: 21.

[33] World Health Organization Syrian Arab Republic. From Preparedness to Vaccination: WHO Syria - Special COVID-19 Report. ReliefWeb. 2022. Available: https://reliefweb.int/report/syrian-arab-republic/preparedness-vaccination-who-syria-special-covid-19-report. Accessed: 28 February 2022.

